# Integrated Droplet-Based Microextraction with ESI-MS for Removal of Matrix Interference in Single-Cell Analysis

**DOI:** 10.1038/srep24730

**Published:** 2016-04-29

**Authors:** Xiao-Chao Zhang, Zhen-Wei Wei, Xiao-Yun Gong, Xing-Yu Si, Yao-Yao Zhao, Cheng-Dui Yang, Si-Chun Zhang, Xin-Rong Zhang

**Affiliations:** 1 Beijing Key Laboratory for Microanalytical Methods and Instrumentation, Department of Chemistry, Tsinghua University, Beijing 100084, China; 2 National Institute of Metrology, Beijing 100013, China

## Abstract

Integrating droplet-based microfluidics with mass spectrometry is essential to high-throughput and multiple analysis of single cells. Nevertheless, matrix effects such as the interference of culture medium and intracellular components influence the sensitivity and the accuracy of results in single-cell analysis. To resolve this problem, we developed a method that integrated droplet-based microextraction with single-cell mass spectrometry. Specific extraction solvent was used to selectively obtain intracellular components of interest and remove interference of other components. Using this method, UDP-Glc-NAc, GSH, GSSG, AMP, ADP and ATP were successfully detected in single MCF-7 cells. We also applied the method to study the change of unicellular metabolites in the biological process of dysfunctional oxidative phosphorylation. The method could not only realize matrix-free, selective and sensitive detection of metabolites in single cells, but also have the capability for reliable and high-throughput single-cell analysis.

Droplet-based microfluidics could manipulate tiny droplets to perform fast^1^,^2^, low-consuming^3^,^4^,^5^, and high-throughput assays^6^,^7^,^8^,^9^, which is suitable for single-cell analysis. Recently, many systems that integrate droplet-based microfluidics and fluorescent probes have been successfully applied in this field^10^,^11^,^12^. Due to the limitation of spectral bandwidth, it is hard to detect multiple components simultaneously for fluorescent probes^13^. In comparison, mass spectrometry is a label-free technique that could achieve simultaneous detection of various components^14^, even provide the structure information of unknown molecules. Thus, it is essential to integrate droplet-based microfluidics with mass spectrometry for multiple analysis at single-cell levels.

Some research works based on integrating these two techniques have been reported. One of them was named as Liquid Extraction Surface Analysis (LESA), which integrated droplet extraction with Nano-ESI for the analysis of blood spot^15^, tissue section^16^, and bacteria colonies^17^. To the best of our knowledge, however, this system has not achieved single-cell analysis since the volume of the droplet used in the technique was at microliter to nanoliter levels, while the volume of typical mammalian cell is only about 1 pL^18^. The cellular components would be diluted too much to be detected if using the droplet with the volume above nanoliter.

Some methods based on ESI-MS have been successfully applied to single-cell metabolite analysis^19^,^20^,^21^. Cellular metabolites were often sampled by metal needles^22^,^23^ or microcapillaries^24^,^25^,^26^,^27^,^28^. Nevertheless, the matrix interference such as the interference of culture medium shouldn’t be ignored in single-cell MS analysis, because culture medium usually contains many molecules similar or identical to cellular components^18^. Besides, the cellular matrix is complex^29^, and contains many components such as proteins, lipids, nucleotides, inorganic salts, *et al.*^18^,^20^. They may interfere each other in the detection, which influences the detection sensitivity of concerned components^30^. Our group has previously proposed a technique^31^ for reducing salt interference in detection of organic biomolecules. However, this technique was incapable to remove the interference among different organic components.

In this study, we proposed a method to integrate droplet-based microextraction with ESI mass spectrometry (see Fig. 1). A pre-loaded micro droplet (about 2 nL) was extruded from a pulled glass capillary’s tip to wrap single cell and extracted the cellular compounds. The extract was sucked back to the capillary’s tip, and detected by ESI-MS after evaporating and redissolving with a small-volume assisted solvent (20 ~ 100 pL). This method had three obvious advantages. First, the use of specific extraction solvent could selectively obtain concerned components and remove interference of other cellular components. Second, without too much sample dilution, this method could not only improve detection sensitivity, but also obtain stable and reliable detection results of single cells. Third, with better mechanical design, the method had the ability for high-throughput single-cell analysis, which would further promote the development of single cell research. Using this method, we have detected UDP-Glc-NAc, GSH, GSSG, AMP, ADP and ATP in single MCF-7 cells, and we also applied this method to study the biological process of dysfunctional oxidative phosphorylation.

## Results and Discussion

### Study of Extraction Solvent

Since the use of different extraction solvent would selectively extract different cellular compounds, we investigated the extraction results of various solvents. We used population cells instead of single cells to perform this experiment, because there is heterogeneity in single cells^32^, which means every cell is different^33^. We used different solvents (acetonitrile, DMSO, water and 25% methanol aqueous solution) to extract parallelly-passaged cells. Ammonium formate solution (150 mM) was used to wash cells at 4 °C. The purpose of using low temperature was to inhibit the enzyme activity such as ATP-hydrolysing enzymes^34^ and weaken biochemical reactions in cells, which prevented the change of metabolites in the pretreatment process. Each dish of MCF-7 cells was added in 1 mL solvent and the cellular compounds was extracted for 20 min. A long extraction time was chosen to ensure cellular compounds were extracted adequately.

The extract of each solvent was detected by a home-made ionization source^35^ (more details of the ionization source were shown in Supplementary Figure S1). The detection results were shown in Fig. 2. The extract of acetonitrile had many peaks between m/z 730 and 850 such as m/z 767, 795 and 821 (see Fig. 2a), and they could be deduced as lipids according to the similar results of other works^35^,^36^,^37^. And the detection result of acetonitrile solvent was shown in Supplementary Figure S2 as blank control. Similar to acetonitrile, DMSO could extract lipids. However, GSH (m/z 306) and UDP-Glc-NAc (m/z 606) were also extracted by DMSO, as shown in Fig. 2b. Different from acetonitrile and DMSO, water could extract GSH, AMP (m/z 346), ADP (m/z 426), ATP (m/z 506), UDP-Glc-NAc and GSSG (m/z 611), but no obvious lipid’s peaks were observed, as shown in Fig. 2c. Among these three kinds of solvent, the order of polarity is acetonitrile < DMSO <water. Lipids of weak polarity could be extracted by acetonitrile instead of water. On the contrary, GSH, AMP, ADP, ATP, UDP-Glc-NAc and GSSG could be extracted by water instead of acetonitrile because their polarities matched better. The polarity of DMSO was between acetonitrile and water, so it could extract lipids and part of those six molecules. Hence, Water was more proper to extract cellular GSH, AMP, ADP, ATP, UDP-Glc-NAc and GSSG, and it could also remove interference of lipids.

**Figure 1:**
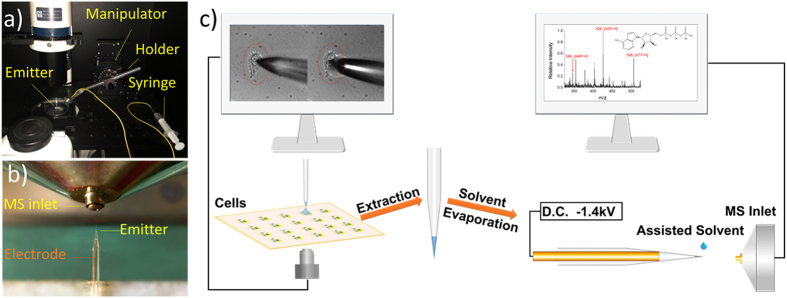
Systems of sampling unicellular metabolites and detection. (**a**) Photograph of sampling platform. A pulled silica glass capillary (Emitter) was fixed on the plastic holder, and the holder was settled in the manipulator. The emitter was linked with a syringe to extrude or suck back the extraction solvent. (**b**) Home-made ion source. Emitter was placed 5 mm in front of the MS inlet. A copper wire was inserted into the emitter as electrode. The distance between electrode and emitter’s tip was 5 mm to ensure the electrode and sample are contactless. (**c**) Schematic of our method. First, the prepared cells were placed under an inverted microscope for sampling. A pulled emitter was pre-loaded about 2 nL extraction solvent (25% methanol aqueous solution, v/v). Second, a three dimensional manipulator was used to precisely place the tip of emitter close to the surface of single cell. And the solvent was extruded onto the cell by a syringe. The cell was wrapped by the solvent for 10 s to extract the cellular compounds, and then the solution was sucked back to the emitter. Third, the obtained extract was let to dry. A drop of assisted solvent (20 ~ 100 pL, 50% methanol aqueous solution, v/v, containing 0.1% ammonium hydroxide) was used to redissolve the biomolecules, and home-made ionization source was used for subsequent detection. The electrode voltage was −1.4 kV for negative mode analysis.

**Figure 2:**
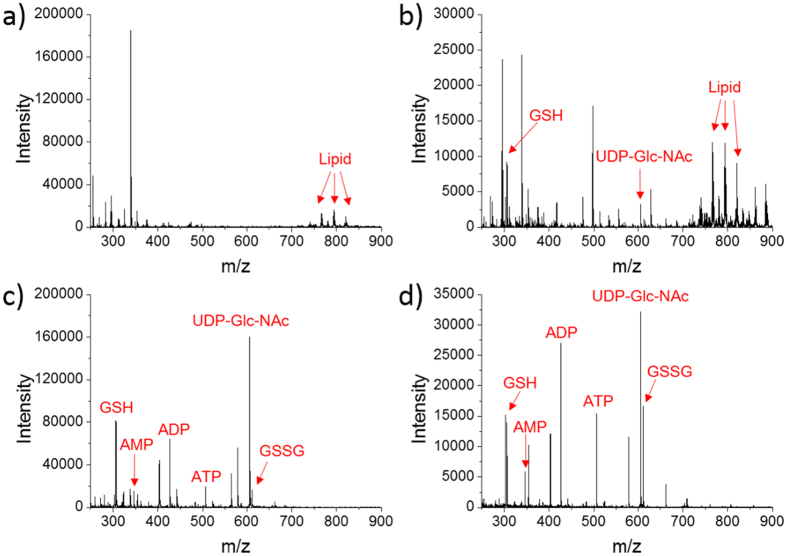
Investigation of extraction solvents by extracting whole dish of MCF-7 cells. MS detection was performed under negative mode. (**a**) Acetonitrile was used as extraction solvent and different lipids were detected. (**b**) DMSO was used as extraction solvent, which could extract GSH and UDP-Glc-NAc besides lipids. (**c**) Water was used as extraction solvent, and GSH, AMP, ADP, ATP, UDP-Glc-NAc and GSSG were extracted. (**d**) 25% methanol aqueous solution (v/v) was used as extraction solvent, GSH, AMP, ADP, ATP, UDP-Glc-NAc and GSSG were extracted.

Because the single-cell treating process contains the step of evaporating extraction solvent (see Fig. 1c), we also tried using 25% methanol aqueous solution to extract cells considering accelerating the evaporating rate. As Fig. 2d shows, those six kinds of molecules are also extracted by 25% methanol aqueous solution. And the detection result of blank control was shown in Supplementary Figure S2. Thus, 25% methanol aqueous solution had the similar extraction effect to water, and it was more suitable for our single-cell experiments because of quicker evaporating rate.

**Figure 3:**
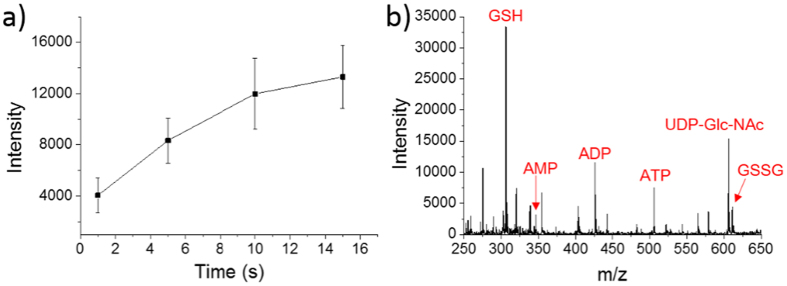
Optimization of unicellular extraction time and the detection result of single MCF-7 cells. (**a**) Single MCF-7 cells were extracted with different time, and the vertical axis represents the MS intensity of ADP (m/z 426). (**b**) The mass spectrum of unicellular metabolites.

To choose the proper single-cell extraction time, we used droplets to extract ADP in single MCF-7 cells with the extraction time of 1 s, 5 s, 10 s and 15 s. As shown in Fig. 3a, the intensity of detected ADP increased gradually along with the extraction time. However, when the droplet covered single cell, the solvent itself was gradually evaporating. If the extraction time is longer, it is more likely to happen that the solvent may totally evaporate before being sucked back into the emitter. Therefore, considering the balance between extraction effect and time cost, 10 s was suitable for our single-cell experiments.

### Single-cell detection

Under the optimized conditions, the obtained mass spectrum of single MCF-7 cells was shown in Fig. 3b. It is obvious that GSH, AMP, ADP, ATP, UDP-Glc-NAc and GSSG have been detected in single cells. The S/N of GSH, AMP, ADP, ATP, UDP-Glc-NAc, and GSSG were 157, 15, 54, 36, 72 and 21, respectively.

**Figure 4:**
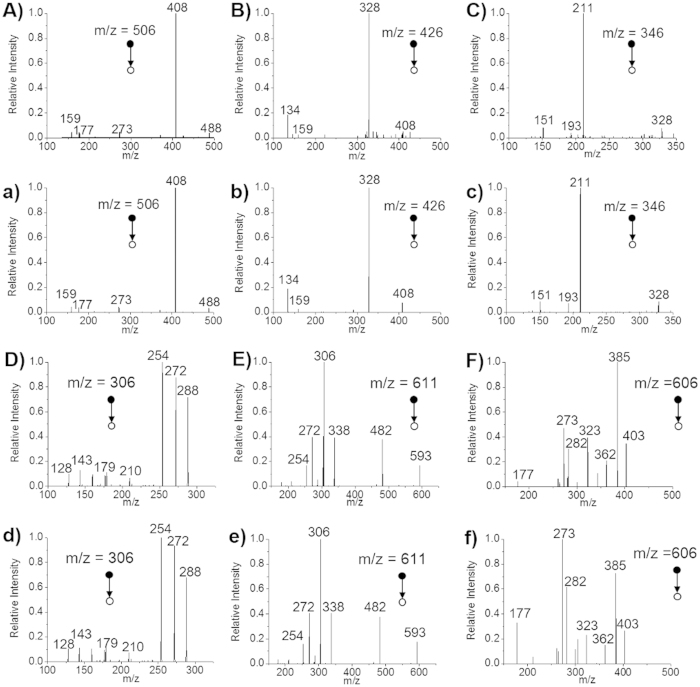
MS/MS spectra of ATP, ADP, AMP, GSH, GSSG and UDP-Glc-NAc. (**A**–**C**) ATP, ADP and AMP in MCF-7 cells. (**a**–**c**) Standard solutions of ATP, ADP and AMP with the concentration of 10 μM. (**D**–**F**) GSH, GSSG and UDP-Glc-NAc in MCF-7 cells. (**d**,**e**) Standard solutions of GSH and GSSG with the concentration of 10 μM. (**f**) MS/MS spectrum of UDP-Glc-NAc from MassBank database (http://www.massbank.jp).

To further validate the results, the MS/MS spectra of them were detected and compared with standard reagents or standard spectrum of database (MassBank database, http://www.massbank.jp). The MS/MS spectra were shown in Fig. 4. Highly-consistent fragmentation rules were observed. For example, in Fig. 4A,a, m/z 408 indicates ATP lost a phosphoric acid unit (H_3_PO_4_, 98 Da) after rearrangement. Further losing adenine (C_5_H_5_N_5_, 135 Da) produced the peak of m/z 273. On this basis, losing D-ribose residue (C_5_H_6_O_3_, 114 Da) generated the peak of m/z 159. And m/z 177 indicates the pyrophosphate anion ([H_3_P_2_O_7_]^−^) that was produced by losing adenosine residue (C_10_H_12_N_5_O_6_P, 329 Da) from ATP.

To objectively evaluate the stability of our method, we calculated the RSD of three important operation steps: pulling the emitter, sucking the solvent, and MS detection. The RSD of inner diameters of emitter’s tip is 9.5%. The RSD of volumes of sucking solvent is 7.3%. And the RSD of MS intensity when detecting the same solution (25% methanol) is 13.4%. More details were shown in Figure S3 in Supplementary Information.

**Figure 5:**
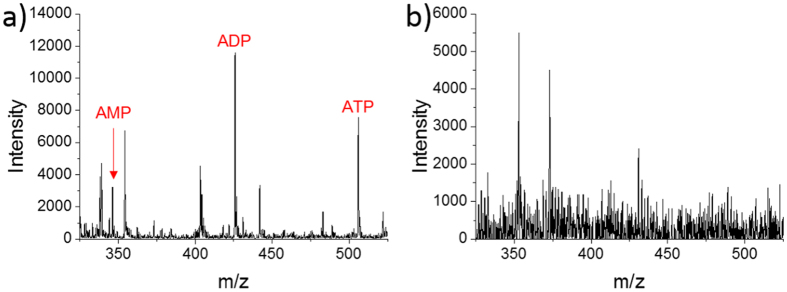
Mass spectra of single MCF-7 cells obtained by different methods. (**a**) Dried single cell was treated and detected by our method. The data range of m/z 325 ~ 525 was chosen to plot. (**b**) Emitter was pre-loaded 2 nL assisted solvent (50% methanol containing 0.1% ammonium hydroxide) and was used to suck cytoplasm of single cell *in situ*. Nano-ESI was used for detection.

To validate our method has the advantage of removing matrix interference, we performed a contrast experiment. We used emitters to suck cytoplasm of single MCF-7 cells with the presence of culture medium, and then using Nano-ESI for detection. The mass spectra obtained by this method and our method were shown in Fig. 5. Unicellular ATP, ADP and AMP were successfully detected by our method because matrix interference was well-removed, as shown in Fig. 5a. The use of droplet-based microextraction could selectively obtain targeted metabolites such as ATP, ADP and AMP, which could also avoid the interference of other cellular organic components such as lipids. In addition, the technique named desalting by crystallization^31^ was used in our method (the steps of evaporating and redissolving the extract). It could also help reducing matrix interference. However, ATP, ADP and AMP couldn’t be detected in the control experiment, as shown in Fig. 5b. It was inevitable to suck in culture medium when sucking the cytoplasm, and the strong matrix interference caused by viscous culture medium might suppress the molecule ions of ATP, ADP and AMP. In comparison, our method is more suitable for detecting targeted metabolites in single cells.

### Drug stimulation towards single cells

A significant application of high-throughput single-cell analysis is drug screening. To illustrate that our method could be used for drug research, we performed drug-stimulation tests. Since dysregulation of oxidative phosphorylation caused by uncoupling is closely related to many human diseases including Parkinson’s disease^38^, heart disease^39^, and obesity^40^. We treated cells with uncoupler Carbonyl cyanide 3-chlorophenylhydrazone (CCCP) to study the change of unicellular metabolites under the state of dysregulation. Different concentrations (2.5 μM, 5.0 μM, 7.5 μM and 10 μM) were used to stimulate MCF-7 cells for 30 min. Then, our method was used for subsequent sampling and detection.

**Figure 6:**
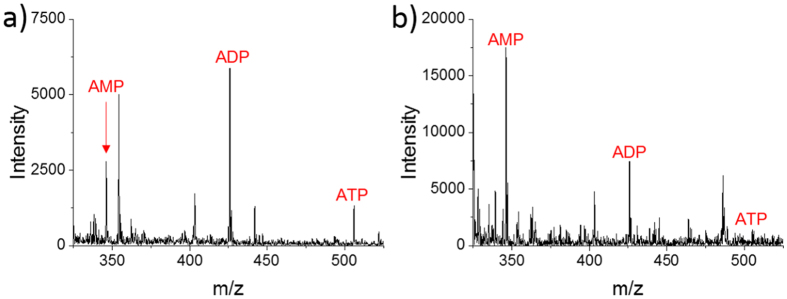
Mass spectra of single MCF-7 cells dosed with CCCP. (**a**) 5.0 μM CCCP was used to treat cells. (**b**) 10 μM CCCP was used to treat cells.

The typical mass spectra were shown in Fig. 6. To avoid the errors caused by variation of emitters, ratios of metabolites were studied in our experiment. In comparison with mass spectrum of untreated single MCF-7 cell (Fig. 5a), significant changes in the relative intensity of MS peaks have been observed. In Fig. 5a, ADP had the highest intensity, ATP was lower, and the intensity of AMP was the lowest. However, when cells were dosed with 5.0 μM CCCP, the relative intensity of ATP became the lowest (Fig. 6a). When 10 μM CCCP stimulated cells, ADP and ATP decreased obviously, and AMP had the highest intensity, as shown in Fig. 6b.

**Figure 7:**
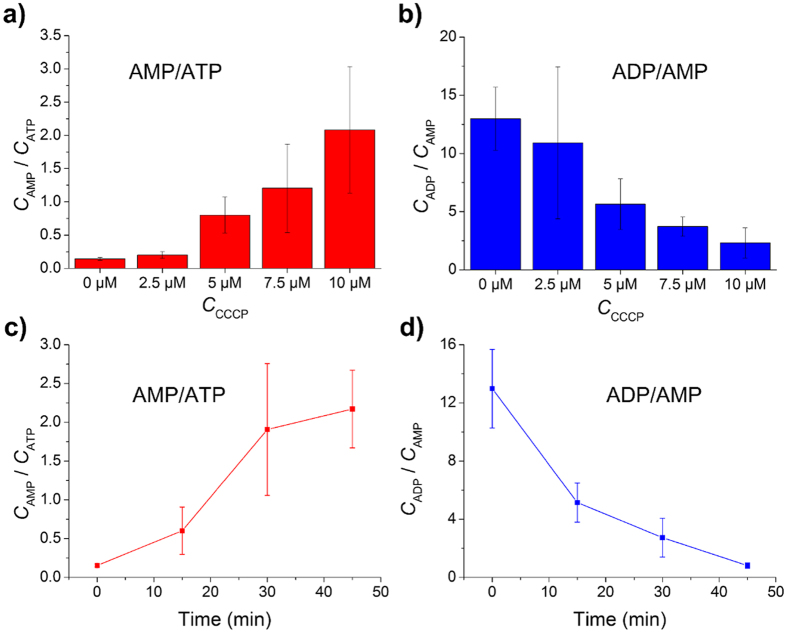
Influence of CCCP concentration and treating time towards single MCF-7 cells. (**a**) Change of AMP/ATP concentration ratios with different drug concentration. Cells were dosed with CCCP for 30 min. (**b**) Change of ADP/AMP concentration ratios with different drug concentration. Cells were dosed with CCCP for 30 min. (**c**) Change of AMP/ATP concentration ratios with different dosing time. Cells were treated with 10 μM CCCP. (**d**) Change of ADP/AMP concentration ratios with different dosing time. Cells were treated with 10 μM CCCP.

Because the relative intensity of MS peaks couldn’t show the exact relative concentration of metabolites, we established standard curves to convert the intensity ratios of AMP/ATP and ADP/AMP into concentration ratios. The standard curves were made by detecting standard reagents with different concentration ratios, more details were introduced in Supplementary Figure S4. After calculation by standard curves, the concentration ratios of AMP/ATP and ADP/AMP in single MCF-7 cells were shown in Fig. 7. It is obvious that the concentration ratio of AMP/ATP grows higher with the increasing of CCCP concentration in Fig. 7a. The concentration of AMP was less than a half of ATP when cells were treated with 2.5 μM CCCP, which was similar to the results of cells without drug treatment. The concentrations of AMP and ATP became approximate when cells were dosed with 5.0 μM CCCP. And when cells were treated with 7.5 μM and 10 μM CCCP, the concentration of AMP became higher than ATP. The result could be explained by the uncoupling effect of CCCP. Along with the increasing of drug concentration, the inhibition to ATP synthesis was stronger^41^,^42^. The uncoupling effect of CCCP weakened the transformation of ADP to ATP, which would increase the concentration of ADP. However, the concentration ratio of ADP/AMP decreased obviously with the increasing of CCCP concentration, as shown in Fig. 7b. The result indicated that the increased concentration of AMP was more than that of ADP, although the absolute concentration of ADP was larger than AMP (the concentration ratio of ADP/AMP was always larger than 1).

We also studied the influence of dosing time towards unicellular metabolites. We chose 10 μM as the concentration of CCCP in order to make the phenomenon more obvious. As shown in Fig. 7c, the concentration ratio of AMP/ATP increased with treating time. The increasing rate during 30 ~ 45 min was apparently lower than that during 15 ~ 30 min. Different from AMP/ATP, the concentration ratio of ADP/AMP decreased with treating time, as shown in Fig. 7d. The decreasing rate became gradually slower.

To validate the reliability of our detection result, we also repeated the drug experiments for population cells. The obtained results were shown in Figures S5 and S6 in Supplementary Information. And consistent rules could be found between population cells and single cells. Besides, the results of our unicellular experiments were also similar to other researches of population cells^41^,^42^ and animals^43^, which validates that our detection result is reliable and our method could be well applied to drug research.

## Conclusion

We have developed a method that integrated droplet-based microextraction with ESI-MS for single-cell analysis. Different cellular compounds could be selectively extracted by various extraction solvents. This method could not only remove matrix interference to increase detection sensitivity, but also obtain stable and reliable detection results (RSD <15%). In our work, 25% methanol aqueous solution was suitable to extract intracellular UDP-Glc-NAc, GSH, GSSG, AMP, ADP and ATP. We also applied this method to detect unicellular metabolites under the state of uncoupled oxidative phosphorylation, and change of ATP, ADP and AMP were observed. Apart from the present work, more improvements could be done in the future study. For example, automated program control could be used to achieve rapid and high-throughput detection of single cells. In addition, quantitative methods such as isotope dilution could be introduced to realize quantification of unicellular metabolites. It is expected that the method could be a useful tool in single-cell analysis.

## Methods

### Reagents and Materials

Adenosine 5′-triphosphate (ATP) disodium trihydrate, Adenosine 5′- diphosphate (ADP) disodium dihydrate and Adenosine 5′- monophosphate (AMP) disodium salt were purchased from Amresco. Reduced L-Glutathione (GSH), Oxidized L-Glutathione (GSSG), Carbonyl cyanide 3-chlorophenylhydrazone (CCCP), Dimethyl sulfoxide (DMSO), HPLC-grade methanol, ethanol and acetonitrile were purchased from Sigma Aldrich. Ammonium formate was purchased from Alfa Aesar. Ultrapure water was obtained from a Millipore water purification system (Barnstead Nanopure, Thermo Scientific, resistivity ≥18 MΩ·cm). All materials used in cell culturing were purchased from Corning (NY, USA), unless otherwise noted.

10 mM stock solution of CCCP was prepared in DMSO and diluted to different concentrations in the cell culture medium according to further experimentation. Solutions of ATP, ADP, AMP, GSH, GSSG and ammonium formate were prepared in ultrapure water. All these solutions were 10 μM and stored at −20 °C except ammonium formate (150 mM, 4 °C).

### Cells

MCF-7 (human breast cancer cell) cell lines were purchased from ABGENT. Cells were cultured in Dulbecco’s modified eagle medium (DMEM) with 10% fetal bovine serum (FBS) and 1% penicillin-streptomycin at 37 °C in a humidified incubator containing 5% CO_2_. Cells were passaged by trypsinization every 2 ~ 3 days when they reached 85 ~ 90% confluence in a 6 cm culture dish. MCF-7 cells used in experiments of oxidative phosphorylation were treated by CCCP with different concentrations and time.

### Emitter Preparation

Emitters used for sampling and MS experiments were pulled from silica glass capillaries using a micropipette puller (P-2000, Sutter Instrument, Novato, CA). Inner diameter of the emitter tip was 3 μm. The diameter was measured under microscope with scale, as shown in Supplementary Figure S3. The parameters of P-2000 program were set as follows: Heat = 950, FIL = 10, VEL = 50, DEL = 145, PUL = 50.

### Sampling and Detection

Single cell sampling experiments were all performed with the use of a three-dimensional manipulator (MP-225, Sutter Instrument) and an inverted microscope (DX30, Dayueweijia Science and Technology Co. Ltd., Beijing), as shown in Fig. 1. Cellular metabolites of single MCF-7 cells were extracted and detected as the procedure shown in Fig. 1c. After drug treatment, cells were quickly washed three times by ammonium formate solution (4 °C) in order to remove culture medium and weaken the biochemical reactions in cells. Then, cells were dried in a vacuum drying oven for 10 minutes. Later, the dried cells were placed under an inverted microscope for sampling. The emitter was pre-loaded about 2 nL extraction solvent (25% methanol aqueous solution, v/v). The volume of extraction solvent was measured and calculated under microscope, as shown in Supplementary Figure S3. A three dimensional manipulator was used to precisely place the tip of emitter close to the surface of a dried cell. And the emitter’s solvent was extruded onto the cell by a syringe.

The cell was wrapped by the solvent for 10 s to extract the cellular compounds, and then the extract was sucked back to the emitter. The extract was dried in open air to evaporate the solvent. In the meantime, the salts would crystallize out but biomolecules would be deposited in the tip of the emitter. Later, a drop of assisted solvent (50% methanol aqueous solution, v/v, containing 0.1% ammonium hydroxide) was used to bedew the emitter’s tip to redissolve the biomolecules, which sucked in 20 ~ 100 pL assisted solvent. Desorption/ionization was performed subsequently. All measurements were performed more than 3 times.

### Mass Spectrometry

All MS experiments were carried out on LTQ mass spectrometer (Thermo Scientific, San Jose, CA). The parameters of instrument were set as follows: capillary temperature = 275 °C, capillary voltage = 9 V, tube lens voltage = 100 V, maximum inject time = 100 ms, microscans = 1. The commercial ionization source was removed and replaced by a home-made ionization source^35^. The ionization source was achieved by inserting a copper wire (as electrode) into the emitter and ensure the wire and emitter’s tip are contactless (the distance is usually 5 mm). The emitter was placed 5 mm in front of MS inlet. The electrode voltage was −1.4 kV for negative mode analysis. When the emitter’s tip was bedewed by the droplet, the electrospray would be triggered. The contactless way could make the pico-liter sample have spray time of 18 s, which is adequate for full scan and MS/MS scan. The photograph of the ionization source was shown in Fig. 1b. MS/MS analysis was performed with 30 eV collision energy.

## Additional Information

**How to cite this article**: Zhang, X.-C. *et al.* Integrated Droplet-Based Microextraction with ESI-MS for Removal of Matrix Interference in Single-Cell Analysis. *Sci. Rep.* **6**, 24730; doi: 10.1038/srep24730 (2016).

## Supplementary Material

Supplementary Information
