# Supplementary material for: Integrated Droplet-Based Microextraction with ESI-MS for Removal of Matrix Interference in Single-Cell Analysis
Source: Sci Rep. 2016 Apr 29;6:24730. doi: 10.1038/srep24730 (PMC4850364; doi:10.1038/srep24730)
Supplement: Supplementary Information [file srep24730-s1.pdf]

Supplementary Information

Integrated Droplet-Based Microextraction with ESI-MS for Removal of Matrix Interference in Single-Cell Analysis

Xiao-Chao Zhang,[†] Zhen-Wei Wei,[†] Xiao-Yun Gong,[‡] Xing-Yu Si,[†] Yao-Yao Zhao,[†] Cheng-Dui Yang,[†] Si-Chun Zhang,* [†] and Xin-Rong Zhang[†]

[†]Beijing Key Laboratory for Microanalytical Methods and Instrumentation, Department of Chemistry, Tsinghua University, Beijing 100084, China

[‡]National Institute of Metrology, Beijing 100013, China

Table of Contents:

Figure S1. Total Ion Chronogram of sample-electrode contactless ESI-MS.

Figure S2. Blank control: acetonitrile and 25% methanol aqueous solution.

Figure S3. Measurements of extraction solvent in emitters.

Figure S4. Standard curves for calibrating MS intensities.

Figure S5 and S6. Influence of CCCP treating time towards MCF-7 population cells.

Total Ion Chronogram. In home-made sample-electrode contactless ESI-MS, the emitter's tip was bedewed by a drop of assisted solvent after applying voltage. The distance between electrode and sample (in emitter's tip) was 5 mm. The Total Ion Chronogram is plotted in Figure S1. The duration time could maintain about 18 s at -1.4 kV. It is adequate for full scan and MS/MS scan.

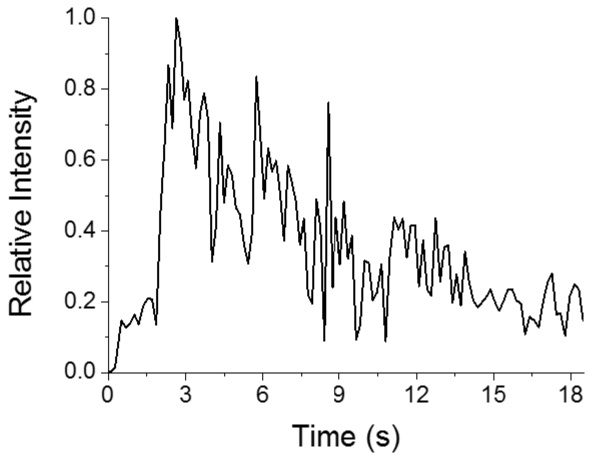

Figure S1. Total Ion Chronogram of sample-electrode contactless ESI-MS.

Blank control of solvents. Acetonitrile and 25% Methanol aqueous solution were detected as blank control in the cellular experiment. The obtained MS spectra were shown in Figure S2.

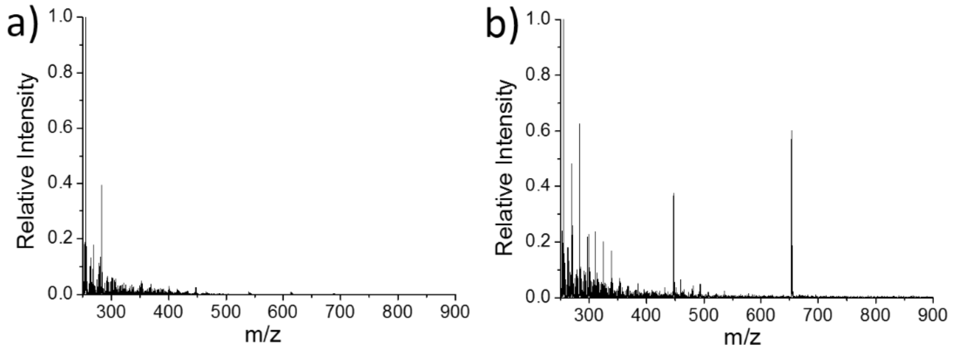

Figure S2. MS spectra of blank control. (a) Acetonitrile. (b) 25% Methanol aqueous solution.

As Figure S2a shows, there are no lipid's peak between m/z 730 and 850 in the acetonitrile such as m/z 767, 795 and 821. There are no metabolite's peaks in Figure S2b, such as GSH (m/z 306), AMP (m/z 346), ADP (m/z 426), ATP (m/z 506), UDP-Glc-NAc (m/z 606) and GSSG (m/z 611). It means the detected metabolites in the cellular experiment were not contamination in solvent or instrument, and our detection results were reliable.

Measurements of extraction solvent in emitters. All measurements were performed under a microscope with scale. The inner diameters of emitter's tips are 3.36, 3.87, 3.72, 3.03 and 3.38 μm, as shown in Figure S3. The average diameter is 3.47 ± 0.33 μm, and RSD is 9.5%. The volumes of extraction solvent were calculated according to the volume formula of frustum cone:

$$V = \frac{\pi}{3} * \mathrm{h} * \frac{d^2 + d * d_1 + d_1^2}{4}$$

h, d, d_1 represent for height of solvent, inner diameter of tip, diameter of bottom.

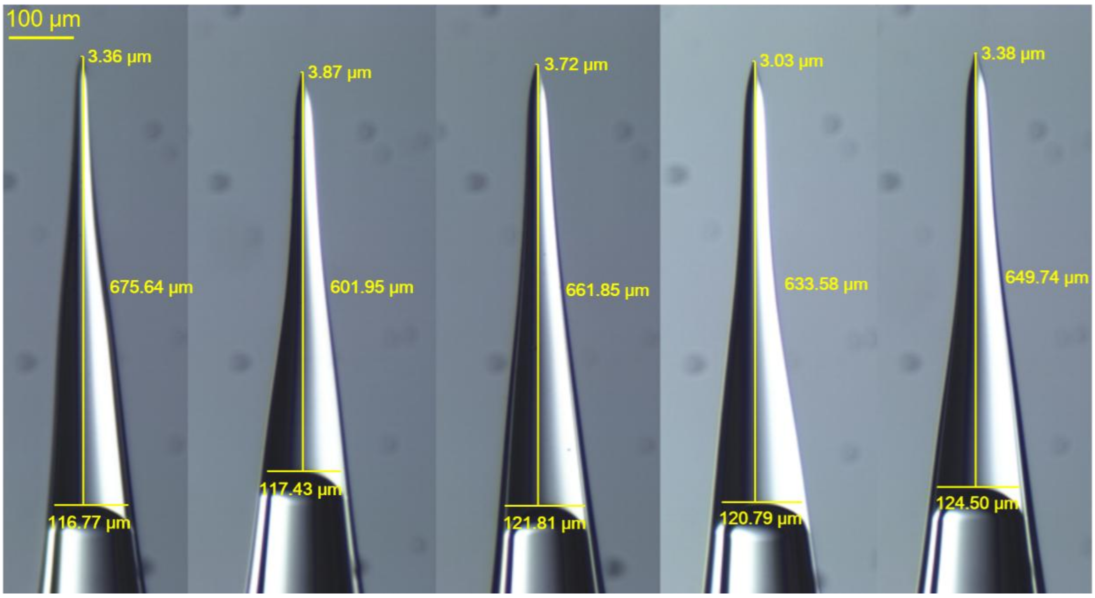

Figure S3. Measurements of volumes of extraction solvent loaded in emitters.

The volumes of extraction solvent are: 2.483, 2.247, 2.652, 2.483 and 2.710 nL. The average volume is 2.466 ± 0.181 nL, and RSD is 7.3%.

The solvents were also detected by our home-made ionization source. The intensities of highest peak (m/z 157) are 2234, 2378, 2165, 1943 and 2776. The average intensity is 2299 ± 309, and RSD is 13.4%.

Standard curves for calculating the concentration ratios of AMP/ATP and ADP/AMP. The standard curves were made by detecting standard reagents with different concentration ratios, as shown in Figure S4.

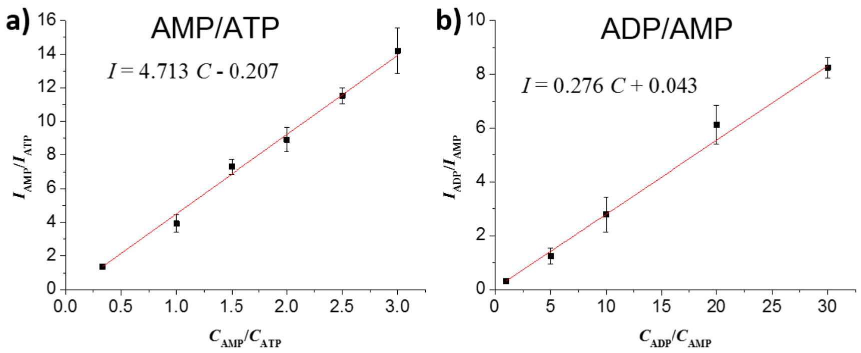

Figure S4. Standard curves for calibrating MS intensities. (a) Standard curves of AMP/ATP. The concentration of ATP was 0.1 μM, the concentrations of AMP were 0.03~0.3 μM. (b) Standard curves of ADP/AMP. The concentration of AMP was 0.1 μM, the concentrations of ADP were 0.1~3 μM.

Influence of CCCP treating time towards MCF-7 population cells. Parallelly-passaged MCF-7 cells were prepared for the experiment, and the cell number of each dish was around 10^6. 10 μM CCCP was added in three culture dishes, and cells were dosed for 15 min, 30 min and 45 min, respectively. A dish of cells without drug stimulation was set as blank control. Then, all dishes of cells were washed by 4 °C ammonium formate solution and dried in a vacuum drying oven. 1 mL 25% methanol aqueous solution was used to extract cells for 20 min. After being filtrated by the filter, the extract was detected by sample-electrode contactless ESI. All measurements were performed more than 3 times. The results were shown in Figure S5.

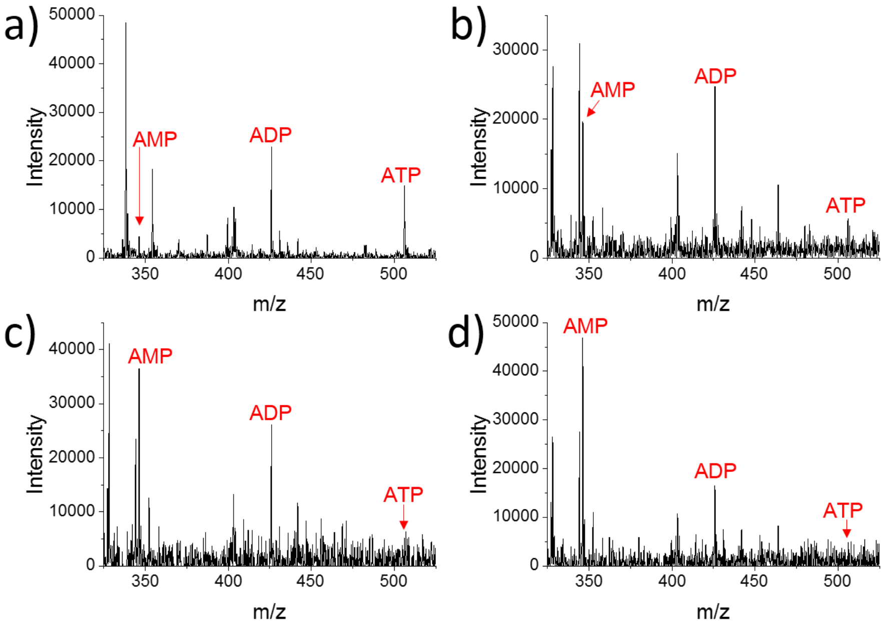

Figure S5. Mass spectra of MCF-7 population cells dosed with 10 μM CCCP. (a) Blank control: cells without drug stimulation. (b) Cells were treated by CCCP for 15 min. (c) Cells were treated by CCCP for 30 min. (d) Cells were treated by CCCP for 45 min.

As Figure S5 shows, after cells were treated by CCCP, the relative intensities of AMP, ADP, and ATP changed a lot. ADP has the highest intensity in them three when cells were not stimulated by drugs. However, along with the drug-treating time, AMP gradually has the highest intensity. The change rules are similar to unicellular detection results. Because the relative intensity of MS peaks couldn't show the exact relative concentration, we also used the standard curves to calculate the concentration ratios of AMP/ATP and ADP/AMP. The calculated results were shown in Figure S6.

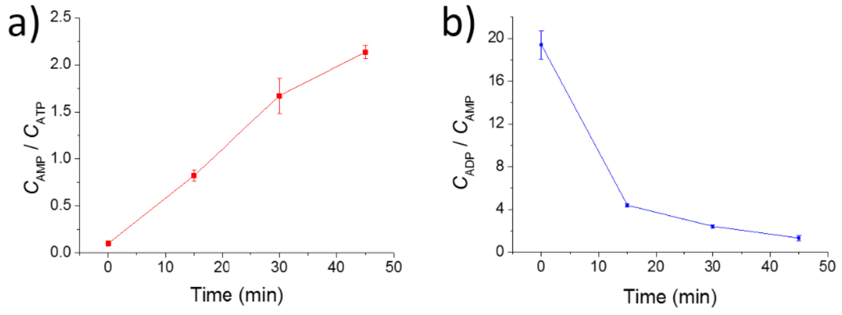

Figure S6. Influence of CCCP treating time towards MCF-7 population cells. Cells were treated

with 10 μM CCCP. (a) Change of AMP/ATP concentration ratios with different treating time. (b) Change of ADP/AMP concentration ratios with different treating time.

In comparison with the unicellular detection results, population cells also showed similar changing rules. For example, the concentration ratio of AMP/ATP increases with treating time (Figure S6a). And the concentration ratio of ADP/AMP decreases with treating time, as shown in Figure S6b.
